# Limited Effects of Endurance or Interval Training on Visceral Adipose Tissue and Systemic Inflammation in Sedentary Middle-Aged Men

**DOI:** 10.1155/2016/2479597

**Published:** 2016-09-29

**Authors:** Joshua H. F. Cooper, Blake E. G. Collins, David R. Adams, Robert A. Robergs, Cheyne E. Donges

**Affiliations:** ^1^School of Exercise Science, Sport and Health, Charles Sturt University, Bathurst, NSW, Australia; ^2^Queensland University of Technology, Brisbane, QLD, Australia

## Abstract

*Purpose*. Limited data exists for the effects of sprint-interval training (SIT) and endurance training (ET) on total body composition, abdominal visceral adipose tissue, and plasma inflammation. Moreover, whether “active” or “passive” recovery in SIT provides a differential effect on these measures remains uncertain.* Methods*. Sedentary middle-aged men (*n* = 62; 49.5 ± 5.8 y; 29.7 ± 3.7 kg·m^2^) underwent abdominal computed tomography, dual-energy X-ray absorptiometry, venepuncture, and exercise testing before and after the interventions, which included the following: 12 wks 3 d·wk^−1^ ET (*n* = 15; 50–60 min cycling; 80% HR_max_), SIT (4–10 × 30 s sprint efforts) with passive (P-SIT; *n* = 15) or active recovery (A-SIT; *n* = 15); or nonexercise control condition (CON; *n* = 14). Changes in cardiorespiratory fitness, whole-body and visceral fat mass, and plasma systemic inflammation were examined.* Results*. Compared to CON, significant increases in interpolated power output (P-SIT, *P* < 0.001; ET, *P* = 0.012; A-SIT, *P* = 0.041) and test duration (P-SIT, *P* = 0.001; ET, *P* = 0.012; A-SIT, *P* = 0.046) occurred after training. Final VO_2_ consumption was increased after P-SIT only (*P* < 0.001). Despite >90% exercise compliance, there was no change in whole-body or visceral fat mass or plasma inflammation (*P* > 0.05).* Conclusion*. In sedentary middle-aged men, SIT was a time-effective alternative to ET in facilitating conditioning responses yet was ineffective in altering body composition and plasma inflammation, and compared to passive recovery, evidenced diminished conditioning responses when employing active recovery.

## 1. Introduction

 Excessive abdominal visceral adipose tissue (VAT) quantity and associated inflammatory cytokine secretion are linked to the development of insulin resistance (IR) and type 2 diabetes (T2D) mellitus [1, 2]. In turn, IR and T2D are precursors to increased atherosclerotic disease progression and end-point cardiovascular disease [3, 4]. Prevalence of obesity, metabolic dysfunction, and consequent disease risk increases with age [5]. Ageing is also associated with sedentary lifestyle and physical inactivity, thus presenting a confluence of factors for the development of whole-body and central obesity in middle-aged populations [6]. A key perception cited for the development of sedentary lifestyle is lack of time to engage in (what is deemed to be) the appropriate volume of physical activity [7].

Recent research indicates that sprint-interval training (SIT) is a time-efficient modality that provides similar and/or comparable health benefits to conventional endurance training (ET) [8–11]. SIT has been shown to increase muscle oxidative capacity [8, 9], cardiorespiratory fitness [10], endurance performance [8–10], and importantly from a disease perspective, insulin sensitivity, and glucose metabolism [11]. It has been suggested that, as is historically observed following ET, SIT may also produce similarly commensurate reductions in whole-body fat mass (FM) and VAT. Further, SIT may prove to be a more time-effective alternative to ET given that most middle-aged individuals do not meet recommended physical activity guidelines [6, 12]; however, studies evidencing the safety and outcome efficacy of SIT in middle-aged men are emerging but remain limited.

While ET is largely a well-researched and accepted modality for the reduction of whole-body FM and abdominal VAT in multiple population groups [13, 14], limited evidence exists to date as to whether SIT can produce similar results to ET. There have been some recent studies which have investigated the effect of interval-type training on central obesity; however, the involved exercise was not maximal (i.e., sprint-type) [15, 16], or there was no inclusion of reference methods to assess abdominal VAT such as magnetic resonance imaging or computed tomography [10, 16, 17]. Despite these shortcomings, in the studies of SIT, there were decreases in measures of central obesity as well as whole-body fat mass [10, 17]. Accordingly, there may be beneficial effects of SIT on abdominal VAT reduction, yet, to the authors knowledge, there has been no study to use reference method VAT analysis of SIT, especially in comparison to conventional ET. Moreover, the current SIT-central obesity data are limited in application to middle-aged adults given that most data pertains to younger trained/recreationally active participants [10, 17]. In addition, some studies include only a SIT condition (i.e., no ET or variation of SIT as a comparison) [10] or a cosex design, thereby introducing potential sex-specific conflict in pooled-sex analysis [10, 17]. Thus, further research is needed in more appropriately examining the effects of SIT on whole-body fat and abdominal VAT.

Importantly, abdominal VAT is a reported site for the release of proinflammatory cytokines, which participate in insulin signal transduction abnormalities and atherosclerotic disease processes [1, 2, 5]. Of the previously studied modalities, ET evidences the greatest capacity for abdominal VAT reduction [13, 14]. That said, ET is the most extensively studied modality, with minimal evidence thus far for SIT. To date, ET appears to produce the most beneficial response in, respectively, decreasing proinflammatory (e.g., tumor necrosis factor-*α* [TNF-*α*], interleukin-6 [IL-6], and C-reactive protein [CRP]) and increasing anti-inflammatory cytokines (e.g., IL-4, and IL-10), collectively facilitating cytokine profile (i.e., balance) towards a more normoglycaemic/atheroprotective state [18–21]. Again, the effects of SIT remain relatively unstudied and poorly understood. A recent study concluded no effect of SIT on plasma systemic inflammation, despite only incorporating a 2 wk intervention period [22]. Regardless of the lack of research for SIT, it appears possible that if SIT elicits VAT reduction, decreases may also be observed in plasma markers of chronic systemic inflammation.

The purpose of this study was to compare 12 wks conventional ET to SIT for effects on whole-body FM, abdominal VAT, and systemic inflammation in sedentary middle-aged men. A further aim was to examine whether additional between-interval ET activity (i.e., passive versus active recovery) would confer a different magnitude (i.e., greater extent) of response on outcome data. We hypothesised that ET would decrease whole-body FM, abdominal VAT, and systemic inflammation to a greater extent than the SIT conditions, with active SIT being more effective than passive SIT.

## 2. Methods

### 2.1. Participants

Sixty-two middle-aged (40–60 y) men volunteered for this study (data in Table 1). Participants were sedentary at baseline, defined as no regular structured or incidental exercise or physical activity >2 d·wk^−1^ in the 12 months priorly. Participants were screened by a physician for preexisting diabetes, cardiovascular disease, renal or hepatic disorders, immunological irregularities, rheumatoid arthritis, periodontal disease, chronic obstructive pulmonary disease, and any other condition linked to systemic inflammatory responses. Volunteers who were tobacco smokers (<1 y cessation), had orthopaedic limitations, or were taking lipid-lowering, antihypertensive, anti-inflammatory, or other potentially confounding medications were not involved in the study. Following recruitment, participants attended an information seminar and received written and verbal information of all study procedures. Participants provided verbal and written informed consent prior to becoming involved. The study was approved by the Institutional Ethics committee and conformed to standards for the use of human subjects in research as outlined in 5th revision of the Declaration of Helsinki.

### 2.2. Study Overview

After prescreening and recruitment, eligible participants attended an information seminar where all procedures were explained and discussed. In a fasted and rested state, participants attended the Institutional Laboratory between 0530 and 0830 h for baseline testing and sequentially completed computed tomography (CT), dual-energy X-ray absorptiometry (DXA), blood pressure assessment, anthropometry, venous blood collection, and a submaximal exercise stress test (GXT). Participants were then randomized into endurance (ET; *n* = 15), “active” recovery sprint-interval training (A-SIT; *n* = 15), “passive” recovery sprint-interval training (P-SIT; *n* = 15), or a nonexercising control condition (CON; *n* = 14). Participants in the exercise groups completed 12 wk, 3 d·wk^−1^ fully supervised, periodized, and progressive programs. After the 12 wk intervention period and in a standardized manner (same time of day, same equipment, same tester, etc.) according to pretesting, participants returned to the laboratories and repeated all baseline test procedures. Participants received no remuneration or incentives for their involvement in the study.

### 2.3. Diet and Physical Activity Control

At the preintervention information and familiarisation seminar and regularly throughout the interventions, all participants were briefed verbally and in writing of the importance of maintaining previous dietary and physical activity habits. Participants were asked to maintain regular food and beverage type, macronutrient content, cooking preparation, portion size, consumption time, and so forth as closely as possible to preintervention dietary patterns during the 12 wk study period. Although sedentary at study baseline, participants in the interventions were asked to maintain their prestudy incidental physical activity habits and to not engage in any additional planned or incidental physical activities during the 12 wk training period.

### 2.4. Graded Exercise Testing

Human ethics, Exercise and Sports Science, Australia, and the American College of Sports Medicine guidelines advocate the need for physician supervision during maximal exercise testing as well as SIT in the recruited cohort given age and risk-factor status. It was not logistically possible to have a physician in attendance at all exercise testing and training sessions. Thus, submaximal GXT and SIT procedures were consequently administered with 85% of estimated heart rate (85%  HR_max_) maximum serving as termination criteria in all study exercise procedures. Blood pressure and electrocardiographic analysis were conducted during the GXT for anomaly identification. Participants were assessed (supervised by RR) for pressure and trace anomalies associated with underlying disease (e.g., hypertensive responses, ST-segment abnormalities, ectopic activity, and bundle-branch blocks) likely to be exacerbated by exercise, as previously recommended [23]. One participant was identified as having concerning anomalies and was excluded; all others were randomized to a study condition.

An electronically braked cycle ergometer (CASE ECG Stress Test System, General Electric Medical Systems, Milwaukee, WI) was used to conduct the GXT. An initial power output of 50 W was followed by increments in power output of 25 W·min^−1^ until 85% of estimated heart rate maximum (HR_max_) was achieved, as identified via a cohort-specific equation (205.9 − 0.685 × age) [24]. Expired gases and flow volumes were collected during the GXT and analysed by a calibrated metabolic cart (TrueOne 2400, ParvoMedics, Salt Lake City, Utah) to provide oxygen uptake measures. The GXT was terminated at the predetermined 85%  HR_max_ value. Interpolated 85% workload (last completed workload + fraction of last attempted workload) and test duration (time to 85%) were recorded to provide intervention outcome data and assist the respective training intervention programs.

### 2.5. Exercise Training Interventions

As exercise session duration is a primary consideration in time cost: benefit for middle-aged cohorts, we opted to provide the advocated guidelines as an upper limit for the ET methods (e.g., 150–180 min) and appropriately progressive SIT programs that were matched to each other for session duration but considerably shorter in duration (50%) than the ET condition. All exercise sessions were directly administered and supervised by the research team (JC and BC), with participant attendance and compliance being documented and monitored throughout. Training was standardized for time of day for each participant and occurred within a climate-controlled laboratory at the Institution. Heart rate, rating of perceived exertion (RPE), cycling duration, pedalling resistance, interval repetitions, and recovery time periods were all monitored and controlled throughout the 12 wk intervention. All exercise group participants received verbal familiarisation with the exercise session structure, physiological demands, and equipment used prior to commencing training and received strong verbal encouragement throughout each session of the program.

#### 2.5.1. Endurance Training (ET)

The ET program consisted of cycle ergometry (828e, Monark AB, Varberg, Sweden) at a workload which elicited ~80–85% of age-predicted HR_max_ or (initially) ~90% of the final workload obtained in the GXT. Training commenced at 50 min·session^−1^ for Wks 1–6 and increased to 60 min·session for Wks 7–12. Mean session Watts, RPE, and heart rate were recorded in each session. As participants conditioning increased, the workload was increased so as to maintain an intensity of ~80–85% of HR_max_.

#### 2.5.2. Passive Recovery Sprint-Interval Training (P-SIT)

The P-SIT training program involved cycle ergometry (828E, Monark Exercise AB, Varberg, Sweden) and included a 5 min (limited) light-moderate warm-up/cool-down of either side of a SIT component. The SIT component is comprised of 1 : 6 work : rest cycles, via 30 s sprint efforts followed by 3 min of passive recovery. Training commenced with four interval efforts, increasing by one effort per fortnight, with 10 efforts being completed at program completion. Pilot testing in an analogous cohort revealed that a cadence of 120 rev·min^−1^ and 5-6% body mass resistance optimized the 30 s work stimulus (e.g., required a “maximal” effort). In the between-interval period, P-SIT participants either remained stationary on the bike or were allowed restricted movement (slow walking; limited in distance) around the training room (60 m^2^).

#### 2.5.3. Active Recovery Sprint-Interval Training (A-SIT)

The A-SIT training program was identical in all program aspects as the P-SIT program (same ergometers, number of efforts, work cadence, progression, etc.); however, it consisted of 3 min (between-interval) periods involving continued cycling at 60 rev·min^−1^ at a lightened resistance (e.g., 1.0–1.5 kp, depending on the participant). Pilot testing confirmed that this combination of cadence and resistance permitted sufficient recovery so as to ensure that participants were to achieve 120 rpm for similar time duration as P-SIT participants during each ensuing interval effort.

### 2.6. Measures

#### 2.6.1. Computed Tomography

At baseline testing, participants firstly arrived at a local radiology facility for computed tomography of the abdominal region. Upon arrival, participants voided the bladder and were positioned as central as possible in the gantry regarding vertex-pubis symphysis alignment. An anterior-posterior scanogram (scout radiograph) of the lower abdomen and pelvis was conducted using a 64-slice multidetector CT (Toshiba Aquilion, Toshiba Medical Systems, Tokyo, Japan). A volume acquisition compartment 77 mm in length was obtained (120 kv, 50 mA, and 0.5 s tube rotation) cephalically from the superior end-plate of L4 during suspended inspiration. After scanning, eleven 7.0 mm contiguous axial images were reconstructed in a maximal display field of view (500 mm) for volume calculation with an attenuation range of −180 to −30 Hounsfield units, and the total adipose tissue (TAT), VAT and subcutaneous adipose tissue (SAT) compartments were determined as described previously [25].

#### 2.6.2. Dual-Energy X-Ray Absorptiometry, Anthropometry and Blood Pressure Assessment

A supine DXA scan was undertaken to estimate whole-body composition (Norland XR800, Cooper Surgical Company, Turnbull, CT, USA). Participants were positioned centrally on the bench of the DXA machine and a whole-body scan was completed with a resolution of 4.5 × 9.0 mm and scanning speed of 130 mm·sec^−1^. The scan was then analysed with customized software (Illuminatus DXA, version 4.2.0, Turnbull, CT, USA) for FM and fat free mass (FFM) which are reported in absolute (kg) and relative (%) terms. All pre- and postintervention scanning procedures and analyses were standardized pre to post (e.g., fasted, rested, no alcohol in the prior 24 h, scan settings, and participant positioning).

Following DXA, measurements of body mass and waist and hip circumference were obtained for each participant. Body mass was taken wearing minimal clothing and standardized before and after intervention. Waist and hip circumference measurements were collected as described previously [26] and by the same tester before/after intervention (CD). Blood pressure was measured via auscultation after ~10 min of rest. Systolic and diastolic pressures were recorded three times with a 1 min rest period between measurements. The lowest paired systolic and diastolic values were recorded.

#### 2.6.3. Venous Blood Collection and Analysis

Participants then underwent venepuncture procedures in which a cannula was inserted into a medial antecubital vein and blood was manually drawn by syringe and gently ejected into prechilled (on ice) tubes treated with ethylenediaminetetraacetic acid and serine protease inhibitor (Pefabloc SC, Sigma-Aldrich, Sydney, Australia) to prevent cytokine degradation. Drawn blood was also ejected into serum separator tubes and left to clot within ~20–30 min. The prechilled and clotted serum tubes were centrifuged (refrigerated) for 10 min at 4°C, and plasma and serum specimens were stored at −80°C until analysis (<2 wks after collection).

Serum was analysed for total cholesterol, high-density lipoprotein cholesterol, triglycerides, glucose, and CRP on a high-throughput automated blood analyser according to the manufacturer and respective kit instructions (Dimension EXL, Siemens Healthcare Diagnostics, Sydney, Australia). The Friedewald equation was used to estimate low-density lipoprotein cholesterol as described previously [26]. Intra- and interassay coefficient of variation (CV) were less than 6.1% for all measured analytes. Commercially available enzyme-linked immunosorbent assays (Jomar Life Research, Melbourne) were used to analyse plasma for IL-6, IL-10, TNF-*α*, and MCP-1. Each participant pre- and postsample was loaded sequentially on the same plate, and all standard curves were *R* ≥ 0.99. Intra- and interassay CV were reported (manufacturer) <10% for all analytes.

### 2.7. Statistical Analysis

A sample size of ≥10 participants per condition was required in order to acquire an effect size of 0.25 at *α* = 0.05 according to* a priori* analysis [27] (G^*∗*^Power v.3). However, >14 participants were involved to help avoid type II error. The subject sample size used in this study is similar to recent studies which have reported significant intervention effects of ET and SIT on measures of body composition [10, 16, 17]. Repeated-measures analysis of covariance (ANCOVA) was used to assess condition × time interactions with baseline data (for each respective variable) used as the covariate. If a main effect was observed in the ANCOVA, one-way ANOVA with Bonferroni's multiple comparison* post hoc* tests applied to determine significant differences. Effect size (ES) data for pre-to-post change and between-group differences were determined using unbiased Hedges Cohen's *d* calculation (trivial: <0.20; small: 0.20–0.49; moderate: 0.50–0.79; or large: >0.80). Statistical significance for the ANCOVA and one-way ANOVA was accepted when *P* < 0.05. ES data was only reported if deemed to have an upper moderate or large effect (*d* > 0.70). All data are presented as mean ± standard deviation (SD).

## 3. Results

### 3.1. Exercise Training Compliance

All subjects attended >30 of the 36 organised exercise training sessions (~91%), with mean session attendance being 32 of the 36 sessions (see Table 2). One-way ANOVA revealed that there was no significant difference in training compliance between groups (*P* > 0.05). While there were no adverse events arising from the exercise training interventions, 3 participants, one from each of the P-SIT, A-SIT, and CON groups, respectively, did not complete the 12 wk interventions (1*n* = orthopaedic limitations; 2*n* = time constraints) and were excluded from analysis, resulting in final numbers: ET (*n* = 15), P-SIT (*n* = 15), A-SIT (*n* = 15), and CON (*n* = 14).

### 3.2. Cardiorespiratory Fitness Measures

A significant main effect was identified for increases in VO_2_ uptake, power output, and GXT time duration at 85%  HR_max_ (*P* < 0.05). One-way ANOVA identified increases for peak output (P-SIT, *P* < 0.001; ET, *P* = 0.009; A-SIT, *P* = 0.05), time duration (P-SIT, *P* = 0.001; ET, *P* = 0.01), and VO_2_ at 85%  HR_max_ (L·min^−1^) (P-SIT, *P* = 0.001) (Table 2). In comparison to CON, at 85%  HR_max_, there was a significant pre-to-post difference by the exercise interventions for increases in power output (P-SIT, *P* < 0.001; ET, *P* = 0.012; A-SIT, *P* = 0.041), time duration (P-SIT, *P* = 0.001; ET, *P* = 0.012; A-SIT, *P* = 0.046), and VO_2_ (L·min^−1^) (P-SIT, *P* < 0.001) (Table 2). Although no significant differences in change of VO_2_ at 85%  HR_max_ (L·min^−1^) were observed between the CON and ET (*P* = 0.063) or CON and A-SIT (*P* = 0.055), between-group ES data showed a trend for difference (ET versus CON, *d* = 0.97; A-SIT versus CON, *d* = 0.81).

### 3.3. Body Composition, Abdominal CT, and Anthropometry

There was no significant difference in anthropometry measures after the interventions (*P* > 0.05) and only trivial to moderate ES data for pre-to-post and between-condition comparisons (all *d* < 0.60). Further, there were no significant changes in FM, FFM, or abdominal CT measures pre-to-post or between conditions (*P* > 0.05) (see Table 3). The ES data indicated only trivial to small (all *d* < 0.22) ES data for changes in FM and FFM. Although a large effect between CON-ET groups presented for FFM increase (*d* = 1.36), ES data for pre-to-post change in abdominal CT were small to trivial (all *d* < 0.41). However, for VAT (cm^3^), a large and moderate ES, respectively, presented between P-SIT and CON (*d* = 0.91) and A-SIT and CON (*d* = 0.70). For VAT (%), a moderate ES presented between A-SIT and CON (*d* = 0.78) and P-SIT and CON (*d* = 0.78).

### 3.4. Biochemistry, Plasma CRP, and Cytokines

There were no significant pre-to-post or between-condition differences of fasting glucose, cholesterol, LDL, HDL or triglycerides (*P* > 0.05). Aside from moderate ES data for a pre-to-post difference in glucose between CON and A-SIT (*d* = 0.77), all pre-to-post (all *d* < 0.57) and between-group (all *d* < 0.58) biochemistry ES data were trivial to moderate. There were no significant pre-to-post or between-condition differences for plasma CRP or studied cytokines (*P* > 0.05; see Table 4). The ES data showed a moderate ES for CRP reduction between P-SIT and ET (*d* = 0.70) and for IL-10 moderate ES for increase between P-SIT and A-SIT (*d* = 0.71). Otherwise, only trivial to moderate ES data were detected for pre-to-post (all *d* < 0.59) and between-group (all *d* < 0.65) changes of CRP and the studied cytokines.

## 4. Discussion

This study compared conventional ET to age/health-status appropriate SIT for respective effects on whole-body composition, abdominal VAT, and pro- and anti-inflammatory cytokine concentrations in a sedentary overweight middle-aged male population. This study also examined whether a passive (P-SIT) or active (A-SIT) recovery period (between-intervals efforts) would facilitate a differential magnitude of response in outcome measures. Specifically, in addition to completing the P-SIT program, the A-SIT group completed further 27–81 mins (range from Wks 1–12) of moderate intensity exercise per week. We incorporated this study design to ascertain whether the coupling of ET with P-SIT (in recovery) offered additive effects to outcome measures. After intervention, we observed significant improvements in fitness measures across all exercise conditions, yet, surprisingly, increases observed in P-SIT appeared more robust in comparison to A-SIT, despite matching of the 30 s interval efforts. Accordingly, while requiring further elucidation, in the studied population, there may be evidence of an inhibitory effect of combining ET with SIT, exacerbated via the absence of sufficient between-effort “recovery” periods. Previously, ET and SIT have both been reported to significantly reduce body fat [10, 16, 17]; however, to date, of ET and SIT, only evidence exists for a positive effect of ET on abdominal VAT [13]. In the studied cohort, our 12 wk intervention did not result in a reduction of FM or abdominal VAT following either of the SIT interventions. In addition, we observed no changes in inflammatory measures following all exercise interventions. Such a response raises further questions regarding the efficacy of exercise training without caloric restriction to facilitate favourable changes in body composition, abdominal VAT, and chronic systemic inflammation.

Accumulating evidence supports that SIT is a time-efficient alternative to conventional ET for the facilitation of musculoskeletal adaptations associated with oxidative metabolism [8–11]. In this study, all three exercise conditions improved measures of cardiorespiratory fitness; however, P-SIT appeared to elicit the greatest relative changes and difference to the CON across each cardiorespiratory fitness measure. These data further support the work of these previous studies and further highlight the role of exercise intensity (compared with duration) in eliciting rapid exercise-induced conditioning-based adaptations. However, the blunted cardiorespiratory fitness improvements observed in A-SIT (when compared to P-SIT) indicate that an inhibitory effect may be present in the A-SIT data. Nader [28] has previously outlined the concurrent training “effect,” wherein the combination of strength and endurance exercise results in potential molecular interference in phenotype-specific adaptive signalling and diminished adaptation in comparison to single-mode completion (e.g., strength or endurance alone). The A-SIT sessions here could be viewed as inducing short-duration high-intensity periods of maximal force with similar duration to 8–10 RM strength-training set, yet combined with continuous moderate intensity endurance exercise in the recovery period. Thus, the A-SIT condition essentially parallels aspects of “Fartlek-”style training, which is ingrained in training programs of many well-conditioned/prepared athletes [29, 30]. Nevertheless, in the studied population group, there may have been a detrimental effect given their lack of condition to accommodate the intense training stimulus. As reported previously, potential inhibitory mechanisms observed as a result of a concurrent training effect include diminished neural activation rapidity, impaired glycogen regulation, muscle fiber-type transformation, ADP-ATP ratio, and associated effect on molecular signalling processes orchestrating muscle protein turnover [28]. Further research may be warranted in investigating these mechanisms in response to A-SIT (compared to P-SIT) in deconditioned middle-aged populations.

Contrary to the findings in this study, SIT and ET have previously been shown to decrease body mass and whole-body FM, thereby positively influencing relative whole-body composition [10, 16, 17]. Accumulating data indicates that 150 min·wk^−1^ of moderate intensity exercise may be insufficient to reduce body mass and may only serve to maintain or ameliorate gain in weight [12, 31]. In our study, the ET group commenced at 150 and progressed to 180 min·wk^−1^ and cycled at a moderate-to-vigorous intensity. The data shows lack of change in whole-body FM and raises questions as to whether middle-aged populations should be increasing session and thus total weekly duration or focusing on optimization of intensity. Accordingly, we sought to appraise an appropriate SIT intervention as an alternative to ET, and, like ET, we observed a limited effect of SIT on whole-body FM. Keating et al. [16] recently reported that moderate intensity cycling ~108–144 min·wk^−1^ over a 12 wk intervention elicited significant reductions in whole-body FM; however, a SIT condition in this study did not alter whole-body or trunk FM. In contrast, prior research has demonstrated that SIT can be effective in reducing whole-body FM [10, 17]. These reductions observed generally appear to be comparable to reductions after ET, yet, in some studies, SIT is reportedly more effective than ET in decreasing whole-body FM [10, 17]. As evidence, Macpherson et al. observed that a SIT protocol of 4–6 repetitions of 30 s maximal running efforts 3 sessions·wk^−1^ for 6 weeks elicited a 12.4% decrease in FM opposed to a 5.8% decrease in a ET group (program range: 90–180 min·wk^−1^). However, this study investigated recreationally active men and women (24 y mean age) and thus whether these age-based and sex-pooled findings apply to overweight middle-aged men remains uncertain. Although data support suggests that SIT may be as effective as ET as a regular exercise alternative [8–11], no current physical guidelines for SIT have been implemented and its effect on health-related outcomes such as whole-body FM are thus far inconsistent and require further elucidation.

Mounting evidence substantiates that the extent (quantity) and dysfunctional regulation (atherogenicity) of abdominal VAT are associated with the extent and progression of metabolic and cardiovascular disease [5, 25]. These findings support why the reduction of VAT currently occupies a central focus in the prevention of these diseases [32]. In this study, there were no differences observed in abdominal VAT in response to the ET or SIT exercise interventions. Historically, ET appears to be the most effective exercise modality for the reduction of abdominal VAT, as shown by a recent meta-analysis and systematic review [13]. Currently, data for the effect of SIT on VAT remain sparse, especially in combination with whole-body FM data. However, similar to our study, others have also reported no effect of SIT on trunk fat (via DXA) [17]. While data are lacking for the effects of SIT on abdominal VAT (CT/MRI-derived) and/or trunk fat (DXA-derived), some interval-type exercise research has reported decreases in measures of central obesity [33, 34]. Hottenrott et al. [33] observed that a high-intensity running-based SIT program (4 × 30 min sessions with 30–120 s intervals) resulted in significant decreases in abdominal VAT; however, these authors used bioelectrical impedance for assessment of abdominal VAT rather than CT or MRI. Furthermore, although theoretically matched for training duration to an ET condition, the SIT condition included greater training frequency and a substantial incorporation of ET, thus raising questions as to whether it was the isolated effect of SIT* per se*. In using similar exercise methodology to our study, Trapp et al. [34] observed a significant reduction in trunk fat mass following a 20 min 3 d·wk^−1^ high-intensity intermittent sprint condition (cycles of 8 s sprint: 12 s recovery). While our interventions found no pre-to-post differences for abdominal VAT, the CON group marginally increased visceral FM. As such, it appears that ET and SIT may have potentially offset gain in VAT across the intervention period, siding with prior research that exercise may serve as a preventative tool against increases in VAT [35].

Equivocal and inconsistent evidence precludes current understanding of the capacity for exercise to decrease markers of chronic systemic inflammation. Some studies report no change in CRP or inflammatory cytokines following exercise intervention [20, 21, 26, 36, 37], while other studies report significant reductions [18–21, 38, 39]. Despite conflicting data to date, in studies where plasma concentrations of CRP and cytokine concentrations reductions are observed, ET appears the more effective modality [18, 19, 21, 39, 40]. The effects of SIT on positively influencing cytokine profile (reducing proinflammatory; increasing anti-inflammatory) are still uncertain. Leggate et al. [22] examined the effect of a 2 wk SIT intervention on inflammation and reported no change in TNF-*α*, IL-6, MCP-1, or IL-10, suggesting that SIT may be relatively ineffective in changing cytokine profile. The limited duration of their intervention would likely have promoted limited/no change in VAT and (theoretically) associated inflammation, which appears to be a consistent finding in other studies [1, 2, 5]. We opted to investigate a longer duration SIT intervention period in order to increase the likelihood of a reduction in VAT and thus associated proinflammatory cytokines (TNF-*α*, IL-6, CRP, and MCP-1). The lack of change in these measures may be explained by the lack of change in abdominal VAT. Further research is needed to verify whether exercise-induced reduction of VAT is essential for reduction of these markers, as our data are inconclusive to show such an effect.

In addition, we examined whether SIT (or ET) may have imparted an anti-inflammatory effect, as would be indicated via increased IL-10 concentrations after training [18, 20, 21, 40]. Despite no significant change denoted in IL-10 for this study, both P-SIT and ET exercise groups evidenced a trend for an increase. However, a large SD, due to large interindividual response variance may have precluded significance. Despite power analyses indicating *n* of 15 per group sufficient to yield statistical significance, and that recent studies have incorporated a similar [16] or smaller [10] sample sizes as this study, a larger sample size in our study may have further substantiated a limited effect of the exercise interventions. In addition, there is existing evidence that 12 wks may induce adaptations which are associated with enhanced metabolic and/or immune regulatory function, yet subsequent effects of these adaptations are not observed until ensuing periods (i.e., at 6 or 12 months). Thus, a limitation of the current study may also be that a longer time frame is required to impart a significant effect on body composition and related plasma inflammatory measures. An additional limitation to our study is that it cannot be ruled out that changes in habitual diet and physical activity may have influenced the study data. In this view, while we strongly emphasised the maintenance of diet and planned/incidental physical activity across the 12 wk period, it is possible that that there may have been some alteration in satiety (in response to the exercise) and/or a decrease in incidental activity given potential training-induced weariness/fatigue from the conducted exercise training sessions.

In undertaking SIT, participants are required to complete supramaximal sprint efforts which are metabolically anaerobic in nature and physically demanding given the involved intensity. Even though, SIT was well-tolerated in this study by all participants, with optimal study compliance and very low study attrition. Accordingly, this method of exercise may be favourable for adoption in the general public, obviously after thorough preexercise medical health screening. Nevertheless, further research is needed to identify a more appropriate means of monitoring the interplay between exercise intensity and cardiovascular work given that, in SIT exercise, participants are undertaking exercise with maximal exertion, yet with delay in the heart rate response. Accordingly, relative submaximal HR_max_ criteria arguably appear redundant given our observation that most participants did not breach 85%  HR_max_ until the conclusion of or immediately after interval.

In conclusion, 12 wks of ET, P-SIT, or A-SIT in an overweight middle-aged male population appeared ineffective in altering whole-body FM, abdominal VAT, or plasma markers underlying chronic systemic inflammation. The lack of change in body composition and abdominal VAT may underline the lack of change in the inflammatory markers. Aligning with previous research, this study has shown P-SIT to promote comparable cardiorespiratory fitness improvements to that of ET. However, it is worth emphasising that the present fitness improvements were based on the use of participant's baseline data with VO_2peak_ as the covariate. However, given that the A-SIT condition presented evidence of a diminished conditioning response in comparison to P-SIT, questions are raised regarding the use of “active” recovery periods in SIT programs involving deconditioned cohorts. Further research may be warranted in examining whether inhibitory mechanisms may be involved in A-SIT versus P-SIT in initially sedentary or deconditioned adults.

## Figures and Tables

**Table 1:** Baseline subject characteristics.

Measure	ET	P-SIT	A-SIT	CON
Age (y)	51.1 ± 5.7	47.2 ± 5.1	49.1 ± 5.3	51.2 ± 7
Height (cm)	177.3 ± 4.4	177.9 ± 8.3	177.0 ± 8.9	176.5 ± 6.3
BMI (kg·m^2^)	30.6 ± 3.4	29.7 ± 4.1	29.7 ± 4.0	28.7 ± 3.6
Waist (cm)	104 ± 9	102 ± 11	100 ± 12	96 ± 9
WHR	1.00 ± 0.06	0.96 ± 0.05	0.97 ± 0.06	0.96 ± 0.06
Total chol. (mmol·L^−1^)	5.6 ± 1.3	5.6 ± 0.8	5.2 ± 1.2	5.8 ± 1.0
LDL chol. (mmol·L^−1^)	3.7 ± 1.2	3.7 ± 0.7	3.6 ± 0.9	3.8 ± 1.0
HDL chol. (mmol·L^−1^)	1.1 ± 0.2	1.1 ± 0.4	1.1 ± 0.3	1.2 ± 0.3
CHR	5.5 ± 1.6	5.7 ± 1.8	4.9 ± 1.5	5.1 ± 1.5
Triglycerides (mmol·L^−1^)	1.9 ± 1.1	1.8 ± 0.8	1.2 ± 0.7	1.7 ± 1.0
Glucose (mmol·L^−1^)	5.8 ± 0.4	5.7 ± 0.5	5.8 ± 0.6	5.4 ± 0.5
SBP (mmHg)	135 ± 12	131 ± 12	136 ± 13	138 ± 15
DBP (mmHg)	86 ± 10	81 ± 9	87 ± 10	92 ± 14

Data are mean ± standard deviation. ET: endurance training condition (*n* = 15); P-SIT: passive sprint-interval training condition (*n* = 15); A-SIT: active sprint-interval training condition (*n* = 15); CON: nonexercising control condition (*n* = 14). BMI: body mass index; WHR: waist to hip ratio; chol: cholesterol; LDL: low-density lipoprotein; HDL: high-density lipoprotein; CHR: cholesterol hazard ratio; SBP: systolic blood pressure; DBP: diastolic blood pressure. One-way ANOVA comparisons showed no differences between conditions for baseline measures (*P* > 0.05).

**Table 2:** Cardiorespiratory fitness measures.

Measure	ET	P-SIT	A-SIT	CON
Study compliance (%)	90.9 ± 7.3	91.2 ± 4.4	91.4 ± 7.6	—
Time GXT (s)				
Pre	373 ± 98	380 ± 90	393 ± 87	389 ± 98
Post	446 ± 109^∧^	470 ± 98^∧^	455 ± 89^∧^	400 ± 96
Raw Δ	73 ± 54^*∗*^	90 ± 41^*∗*^	62 ± 57	10 ± 49
% Δ	21 ± 17	25 ± 14	18 ± 17	3 ± 15
Peak output GXT (W)				
Pre	180 ± 41	183 ± 38	189 ± 36	187 ± 41
Post	211 ± 46^∧^	222 ± 38^∧^	215 ± 36^∧^	191 ± 40
Raw Δ	30 ± 23^*∗*^	39 ± 18^*∗*^	26 ± 24^*∗*^	4 ± 20
% Δ	18 ± 14	23 ± 13	15 ± 14	3 ± 12
VO_2_ GXT (L·min^−1^)				
Pre	2.26 ± 0.46	2.33 ± 0.54	2.61 ± 0.47	2.33 ± 0.49
Post	2.60 ± 0.50	2.82 ± 0.55^∧^	2.91 ± 0.53	2.41 ± 0.40
Raw Δ	0.34 ± 0.28	0.49 ± 0.22^*∗*^	0.30 ± 0.29	0.07 ± 0.26
% Δ	16 ± 15	22 ± 12	12 ± 12	5 ± 13
VO_2_ GXT (mL·kg^−1^·min^−1^)				
Pre	23.6 ± 4.2	24.8 ± 4.8	28.2 ± 4.0	26.4 ± 6.0
Post	27.4 ± 5.2	29.9 ± 5.1^∧^	31.3 ± 3.8	27.1 ± 4.9
Raw Δ	3.8 ± 3.3	5.1 ± 2.4^*∗*^	3.1 ± 3.2	0.7 ± 3.3
% Δ	17 ± 16	22 ± 11	12 ± 12	4 ± 14

Data mean ± standard deviation. ET: endurance training condition (*n* = 15); P-SIT: passive sprint-interval training condition (*n* = 15); A-SIT: active sprint-interval training condition (*n* = 15); CON: nonexercising control condition (*n* = 14). ^*∗*^Significant within-group change from baseline (*P* < 0.05). ^∧^Significant between-group change (*P* < 0.05). GXT: graded exercise test at 85% of age-predicted heart rate maximum (HR_max_); VO_2_: volume of oxygen uptake.

**Table 3:** Body mass and composition data.

Measure	ET	P-SIT	A-SIT	CON
Body mass (kg)				
Pre	96.2 ± 11.4	94.8 ± 20.2	93.9 ± 18.9	89.4 ± 12.1
Post	95.7 ± 12.4	95.7 ± 21.2	94.1 ± 19.1	89.8 ± 12.2
Raw Δ	−0.5 ± 3.5	0.8 ± 1.6	0.2 ± 1.2	0.5 ± 1.2
% Δ	−0.6 ± 3.9	0.7 ± 1.6	0.2 ± 1.4	0.5 ± 1.4
FFM (kg)				
Pre	70.8 ± 7.5	71.4 ± 10.7	70.9 ± 11.6	67.7 ± 6.6
Post	71.2 ± 7.8	72.8 ± 11.2	71.9 ± 11.6	69.2 ± 6.7
Raw Δ	0.37 ± 1.5	1.5 ± 1.9	1.0 ± 1.6	1.4 ± 1.0
% Δ	0.5 ± 2.2	2.0 ± 2.4	1.4 ± 2.2	2.1 ± 1.3
FM (kg)				
Pre	27.2 ± 8.7	24.8 ± 10.9	24.4 ± 8.7	23.3 ± 7.6
Post	28.2 ± 9.6	25.2 ± 11.8	25.1 ± 9.4	23.5 ± 7.1
Raw Δ	1.0 ± 2.1	0.4 ± 2.0	0.7 ± 1.7	0.2 ± 1.3
% Δ	3.5 ± 9.1	1.3 ± 8.5	2.7 ± 6.5	1.8 ± 7.4
FM (%)				
Pre	27.3 ± 6.5	25.0 ± 5.8	25.0 ± 5.3	25.1 ± 5.4
Post	27.9 ± 7.0	24.8 ± 6.0	25.2 ± 5.4	24.9 ± 4.6
Raw Δ	0.6 ± 1.6	−0.2 ± 1.7	0.2 ± 1.4	−0.2 ± 1.2
% Δ	1.9 ± 6.5	−0.5 ± 7.8	1.0 ± 5.7	0.0 ± 6.4
SAT (cm^3^)				
Pre	2396 ± 843	1952 ± 773	2153 ± 788	1852 ± 719
Post	2415 ± 918	1924 ± 746	2179 ± 873	1886 ± 660
Raw Δ	19 ± 163	−29 ± 113	26 ± 188	34 ± 161
% Δ	0.1 ± 7.5	−1.3 ± 6.3	0.7 ± 7.2	4.2 ± 12.2
VAT (cm^3^)				
Pre	1474 ± 427	1540 ± 596	1353 ± 568	1225 ± 448
Post	1489 ± 447	1482 ± 588	1331 ± 705	1441 ± 569
Raw Δ	16 ± 338	−58 ± 302	−22 ± 367	216 ± 280
% Δ	3.2 ± 23.6	−3.0 ± 14.8	−1.3 ± 20.2	18.4 ± 22.5
TAT (cm^3^)				
Pre	3870 ± 1031	3492 ± 1162	3506 ± 1253	3077 ± 1038
Post	3904 ± 1210	3405 ± 1246	3510 ± 1453	3327 ± 1095
Raw Δ	35 ± 427	−87 ± 238	4 ± 452	250 ± 294
% Δ	0.4 ± 11.8	−3.0 ± 5.8	−0.3 ± 9.7	8.8 ± 10.7
VAT (%)				
Pre	38.8 ± 8.5	44.0 ± 8.4	37.8 ± 7.9	40.3 ± 8.4
Post	38.8 ± 7.4	43.3 ± 6.4	37.0 ± 8.6	43.0 ± 7.6
Raw Δ	0.1 ± 4.8	−0.7 ± 4.8	−0.8 ± 4.5	2.7 ± 5.5
% Δ	1.7 ± 12.8	−0.4 ± 10.6	−1.8 ± 12.2	8.2 ± 13.7

Data are mean ± standard deviation. ET: endurance training exercise group, *n* = 15; P-SIT: passive sprint-interval training exercise group, *n* = 15; A-SIT: active sprint-interval training exercise group, *n* = 15; CON: control group, *n* = 14. FFM: total body fat free mass; FM: total body fat mass; SAT: subcutaneous adipose tissue; VAT: abdominal visceral adipose tissue; TAT: total abdominal adipose tissue. There were no significant pre-to-post or between-condition differences in body composition measures after the study interventions (*P* > 0.05).

**Table 4:** Measures of plasma systemic inflammation.

Measure	ET	P-SIT	A-SIT	CON
CRP (mg·L^−1^)				
Pre	2.8 ± 1.6	2.7 ± 1.3	2.1 ± 1.4	2.1 ± 1.5
Post	2.9 ± 1.6	2.1 ± 0.9	1.9 ± 1.2	2.1 ± 1.6
Raw Δ	0.2 ± 1.2	−0.6 ± 1.0	−0.2 ± 0.9	0.0 ± 0.9
% Δ	11.8 ± 40.9	−12.3 ± 50.7	−1.5 ± 34.6	24.3 ± 118.5
IL-6 (pg·L^−1^)				
Pre	3.16 ± 1.36	3.11 ± 1.04	3.29 ± 0.53	3.27 ± 0.64
Post	3.55 ± 0.75	3.18 ± 0.63	3.57 ± 0.38	3.65 ± 0.81
Raw Δ	0.40 ± 1.03	0.08 ± 1.01	0.28 ± 0.46	0.37 ± 0.77
% Δ	22.8 ± 30.6	10.9 ± 35.5	10.1 ± 14.0	12.9 ± 23.2
TNF-*α* (pg·L^−1^)				
Pre	6.1 ± 1.7	6.8 ± 1.5	6.6 ± 1.8	5.2 ± 1.4
Post	6.8 ± 2.1	6.6 ± 1.9	6.4 ± 1.9	5.7 ± 1.3
Raw Δ	0.7 ± 1.6	−0.2 ± 2.2	−0.2 ± 1.3	0.5 ± 1.5
% Δ	13.7 ± 28.8	1.3 ± 31.9	−1.6 ± 23.1	16.0 ± 33.5
MCP-1 (pg·L^−1^)				
Pre	121 ± 43	137 ± 43	145 ± 46	111 ± 86
Post	121 ± 38	129 ± 33	141 ± 43.02	109 ± 84
Raw Δ	−0.2 ± 33.0	−7.5 ± 43.3	−4.1 ± 34.1	−1.8 ± 61.6
% Δ	7.7 ± 37.9	4.0 ± 41.0	3.1 ± 28.8	25.8 ± 92.2
IL-10 (pg·L^−1^)				
Pre	3.72 ± 2.78	3.77 ± 3.03	3.32 ± 2.32	4.98 ± 2.40
Post	5.31 ± 5.25	5.77 ± 4.59	2.93 ± 1.47	5.35 ± 3.17
Raw Δ	1.59 ± 3.72	2.00 ± 4.08	−0.39 ± 2.00	0.38 ± 1.97
% Δ	53.2 ± 85.2	118.0 ± 218.3	16.7 ± 84.5	8.8 ± 42.6

Data are mean ± standard deviation. ET: endurance training exercise group, *n* = 15; P-SIT: passive sprint-interval training exercise group, *n* = 15; A-SIT: active sprint-interval training exercise group, *n* = 15; CON: control group, *n* = 14. CRP: C-reactive protein; IL-6: interleukin 6; TNF*α*: tumor necrosis factor *α*; MCP-1: monocyte chemoattractant protein 1; IL-10: interleukin 10. There were no significant pre-to-post or between-condition differences in plasma CRP and cytokine measures after the study interventions (*P* > 0.05).
